# Ubiquitous Nature of Fluoroquinolones: The Oscillation between Antibacterial and Anticancer Activities

**DOI:** 10.3390/antibiotics6040026

**Published:** 2017-11-07

**Authors:** Temilolu Idowu, Frank Schweizer

**Affiliations:** 1 Department of Chemistry, University of Manitoba, Winnipeg, MB R3T 2N2, Canada; frank.schweizer@umanitoba.ca; 2 Department of Medical Microbiology and Infectious Diseases, University of Manitoba, Winnipeg, MB R3T 1R9, Canada

**Keywords:** antibacterial, antiproliferative, antitumor, fluoroquinolone, gyrase, immunomodulation, topoisomerase

## Abstract

Fluoroquinolones are synthetic antibacterial agents that stabilize the ternary complex of prokaryotic topoisomerase II enzymes (gyrase and Topo IV), leading to extensive DNA fragmentation and bacteria death. Despite the similar structural folds within the critical regions of prokaryotic and eukaryotic topoisomerases, clinically relevant fluoroquinolones display a remarkable selectivity for prokaryotic topoisomerase II, with excellent safety records in humans. Typical agents that target human topoisomerases (such as etoposide, doxorubicin and mitoxantrone) are associated with significant toxicities and secondary malignancies, whereas clinically relevant fluoroquinolones are not known to exhibit such propensities. Although many fluoroquinolones have been shown to display topoisomerase-independent antiproliferative effects against various human cancer cells, those that are significantly active against eukaryotic topoisomerase show the same DNA damaging properties as other topoisomerase poisons. Empirical models also show that fluoroquinolones mediate some unique immunomodulatory activities of suppressing pro-inflammatory cytokines and super-inducing interleukin-2. This article reviews the extended roles of fluoroquinolones and their prospects as lead for the unmet needs of “small and safe” multimodal-targeting drug scaffolds.

## 1. Introduction

Antibiotics are not made, they are simply discovered, and the discovery process is a mixed bag of profound scientific exploration and/or fortunate coincidences. Our current arsenal is mainly made up of compounds that were derived from natural sources, the very source of their woes, and their semi-synthetic derivatives. Microorganisms often secrete trace amount of antibiotics primarily as warfare agents to kill other bacteria or fungi in the evolutionary struggle to gain an advantage over other species that are competing for the same ecological niche [1]. Nature’s laboratory is therefore a good reservoir of antibiotic scaffolds that have been evolutionarily optimized to suit the physicochemical requirements for activity in microorganisms, especially against Gram-negative bacteria. However, given the need for the organism producing an antibiotic to protect itself against the harmful effects of such an antibiotic and the defensive mechanism evolved by other bacteria to the agent, antibiotic-resistance mechanisms to natural products are presumed to be already present in the bacterial community [2]. This phenomenon significantly shortens the time between when an isolated antibiotic is introduced into the clinic and when full-fledged resistance is observed. It is therefore imperative to develop new molecules that have not been previously encountered by microorganisms.

**Figure 1:**
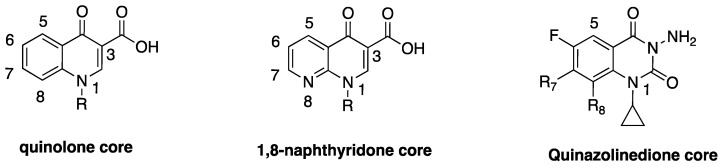
Core structures of the quinolone class of drugs.

Quinolones (Figure 1) are the first class of fully synthetic antibacterial agents to be “discovered”, representing the first set of anti-infective agents that were not modeled knowingly after any natural antibiotics. The scaffold was later optimized to fluoroquinolones (FQs) and has remained an integral part of treating Gram-positive and Gram-negative bacteria infections to date. Only a handful of current antimicrobial agents have broad spectrum of activity across both Gram-positive and Gram-negative bacteria. FQs are a class of privileged antibiotics that enjoy a wide acceptability due to their broad spectrum of bactericidal activity at clinically achievable doses, a wide therapeutic index, comparatively tolerable resistance levels, and their synthetic tractability [3,4]. They act by binding to an intracellular target in the cytosol of bacterial cells where they inhibit the activities of topoisomerase II enzymes (Top II, i.e., gyrase and Top IV), with a high selectivity for prokaryotic enzymes [5]. The inherent physicochemical properties of FQs and their ability to traverse the orthogonal lipid bilayers of Gram-negative bacteria, a feat that some naturally-occurring antibiotics are unable to achieve, is noteworthy. These intrinsic capabilities i.e., intracellular target and suitable physicochemical properties, contributed immensely to the success story of fluoroquinolones and made them desirous as reference scaffold for the development of ideal antibacterial agents.

**Figure 2:**
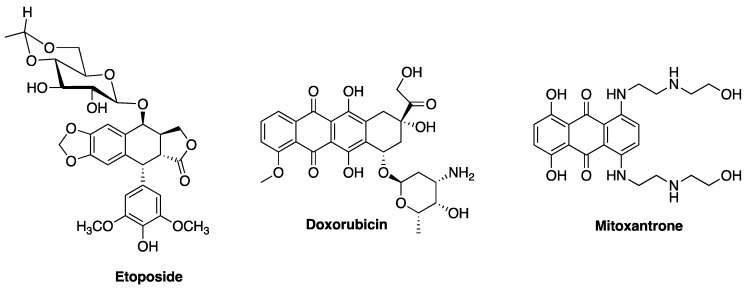
Examples of eukaryotic topoisomerase II poisons.

On the other hand, cancer is a more devastating chronic disease that claims millions of lives every year worldwide [6]. It is a class of disease in which a group of aberrant cells exhibit uncontrollable growth, invade neighboring tissues or organs, and sometimes metastasize [7]. There are several classes of drugs currently in use for managing this disease, but of interest are those that target and act on mammalian topoisomerases. Etoposide (Figure 2) is an antitumor agent that displays a mechanism of action (in eukaryotic cells) that is similar to that of FQs (in prokaryotic cells). They both act on topoisomerases [8,9,10,11]. However, clinically relevant FQs are able to distinguish between bacterial and mammalian topoisomerases, and avoid cross-reactivity with human type II enzyme even at concentrations well beyond their therapeutic doses [5]. Interestingly, some clinically relevant FQs have been shown to be potent antiproliferative agents at much lower concentrations than is needed for their antibacterial activity [12]. It should be noted that FQs that are significantly active against eukaryotic enzymes exhibit the same DNA damaging properties as other topoisomerase poisons [13,14]. Since clinically relevant FQs are known to be safe in humans, how then do they discriminate cancer cells from healthy cells? What can we learn from their physicochemical properties? Moreover, the endowment of quinolones with other “non-classical” biological activities such as anti-HIV-1 integrase [15], cannabinoid receptor-2 agonist/antagonist [16,17], anxiolytic agents [18,19], anti-ischemic activities [20], antiviral effects [21], etc. is continually injecting new enthusiasm towards this scaffold of drug. This article aims to articulate the biological properties of FQs, how they perform their extended roles *viz*-*a*-*viz* their structural basis, and their prospects as a promising drug scaffold.

## 2. Discovery and Development of Fluoroquinolones

Before the discovery of quinolones in the 1960s, antibiotics were majorly sourced from natural products. They were either obtained from plants and animals in the form of host defence peptides that were produced as a part of their innate immunity (e.g., cecropins, defensins, magainins, cathelicidins) [22,23,24,25], or isolated directly from microorganism cultures themselves (e.g., penicillin, aminoglycosides, polymyxins, etc.) [1,26,27]. The accidental discovery of 7-chloroquinolone as an impurity in a distillate during the chemical synthesis of the antimalarial drug, chloroquine, made them the first class of synthetic antibiotics [28]. Although the exact account of how 7-chloroquinolone evolved to 1,8-naphthyridone core (Figure 1) (as in nalidixic acid), and back to quinolone core (as in ciprofloxacin), has been laced with gaps and counter-arguments, George Lesher (the acknowledged discoverer) and his coworkers at Sterling Drugs (now part of Sanofi) are credited as being the first to report the antibacterial activities of this class of drugs in the 1960s [28]. A recent article attempts to shed some lights on the mystery surrounding the discovery process of quinolones, and concluded based on original documents and Lab notes, that Sterling Drugs and Imperial Chemical Industries (now part of AstraZeneca) might have independently discovered this scaffold around the same time [29].

**Figure 3:**
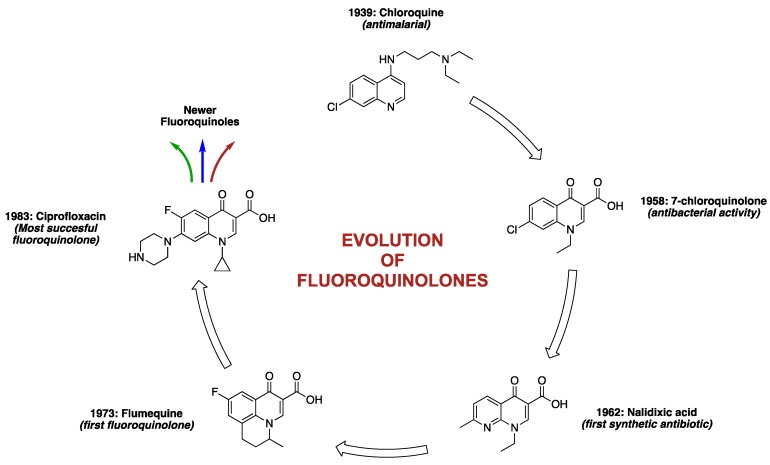
Landmark developmental trends of fluoroquinolones.

Nonetheless, Lesher and coworkers established the anti-Gram-negative bacteria potency of 7-chloroquinolone during biological screening [28], but the compound had limited usefulness due to its high protein binding (approximately 90%) and short half-life (about 90 min) [30]. The lead compound was therefore optimized to nalidixic acid (Figure 3) in 1962, and was used extensively for over 30 years to treat urinary tract infections (UTIs) that are caused by Gram-negative bacteria, mainly *Escherichia coli*. This optimization marked the beginning of an active campaign of chemical synthesis to refine the structure-activity relationship of FQs, such that biological activity and pharmacokinetics could be improved and toxicity and drug interactions diminished [30]. A further introduction of fluorine atom at position-6 of the bicyclic ring system was found to significantly increase tissue penetration, giving rise to the first FQ, flumequine (Figure 3). Quinolone is a generic term that loosely refers to a class of drugs that include quinines, naphthyridines, fluoroquinolones, quinazolines, isothiazoloquinolones, and related agents. FQs (except for enoxacin and gemifloxacin) differ from nalidixic acid in that they have the same quinolone core as the 7-chloroquine impurity, with the addition of a fluorine atom at the sixth position, while nalidixic acid, gemifloxacin, and enoxacin have 1,8-naphthyridone core (Figure 1). The rationale for the back and forth switch in core scaffold is unknown, perhaps due to intellectual property concerns [29], but the addition of fluorine conferred more potent antibiotic action and broader spectrum of activity on this class of drugs [4].

In a bid to further improve the spectrum of activity against Gram-positive species, lower potency, higher frequency of spontaneous bacterial resistance, shorter half-lives, and lower serum concentrations of early FQs [31], several modifications, such as side-chain nuclear manipulations and mono- or bicyclic ring substitutions, were done. The newer FQs were found to exhibit longer elimination half-lives, high oral bioavailability, high potency, extensive tissue penetration, and lower incidences of resistance when compared to the earlier ones [32]. Being a fully synthetic class of drug, the development of FQs has been gradual and systematic (Figure 3). Each generation seems to impart new potencies in a trend that has seen them acquire excellent efficacy towards Gram-positive bacteria in addition to their now optimized potency against Gram-negative bacteria. Remarkably, the newer FQs also display activity against anaerobes [33]. Anaerobes are organisms that do not require oxygen for survival, thereby making drugs that require an oxygen transport into ribosomes (e.g., aminoglycosides) ineffective and never taken up by these organisms [34,35].

Based on their systematic structure optimization process and developmental trends, FQs can be distinctly classified into four generations (Table 1).

## 3. Classical Antimicrobial Activity of Fluoroquinolones

**Figure 4:**
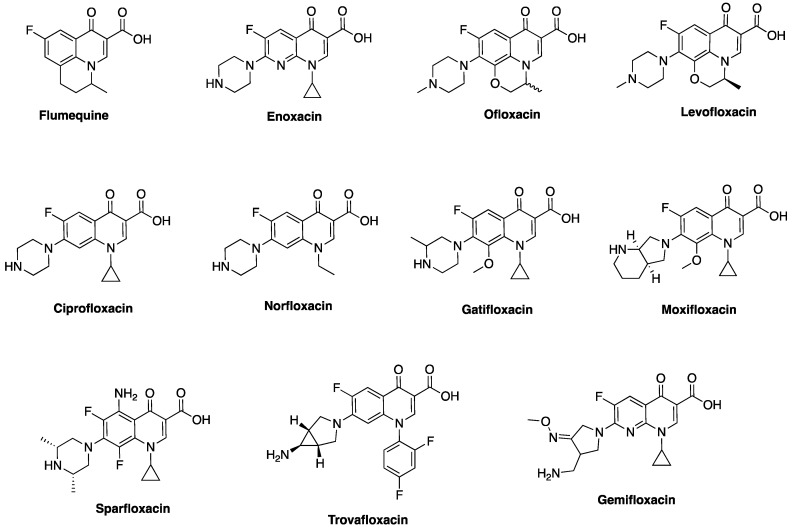
Structures of select fluoroquinolones.

The antimicrobial activity of FQs against a wide range of organisms and their clinical use in the treatment of urinary tract infections (UTIs), prostatitis, bacterial enteric infections, biliary tract infections, sexually transmitted diseases, prophylaxis in immune-compromised neutropenic patients, and a host of other clinical conditions confirm their broad-spectrum of activity [38,39]. While efficacy and spectrum of activity have improved across the generations, tolerability and pharmacokinetic parameters, such as tissue penetration, bioavailability, and serum half-life have also been optimized [40]. The antimicrobial spectrum of the first-generation was largely limited to aerobic Gram-negative bacillary infections, particularly in the urinary tract, while the second generation FQs have enhanced activity (1000-fold) against aerobic Gram-negative and Gram-positive bacteria [41,42]. Ciprofloxacin, a second generation FQ, is the most successful of all FQs to date, both economically and clinically [3]. Newer FQs, such as gatifloxacin, levofloxacin, gemifloxacin, moxifloxacin, etc. (Figure 4) offer enhanced activity against aerobic Gram-negative bacilli and improved Gram-positive activity over ciprofloxacin (e.g., against *Streptococcus pneumoniae* and *Staphylococcus aureus*) [43], but ciprofloxacin and moxifloxacin maintain the best in vitro activity against *Pseudomonas aeruginosa* [43,44]. It is interesting to note that *P. aeruginosa* produces 2-heptyl-3-hydroxy-4(1*H*)-quinolone (PQS), a quorum-sensing signal molecule that shares some core structural features with fluoroquinolones, to regulate numerous virulence genes, including those involved in iron scavenging [45]. In terms of potency, moxifloxacin is more effective against Gram-positive and anaerobes than ciprofloxacin and levofloxacin. Moxifloxacin is often considered as a “respiratory quinolone” because of its significant potency against the respiratory pathogen *S. pneumoniae* [46]. It is currently being investigated as a BPaMZ (bedaquiline + pretomanid + moxifloxacin + pyrazinamide) regimen for the treatment of multidrug resistant tuberculosis, an effort that could shorten the duration of tuberculosis treatment from six to four months if successful [47,48]. When compared to other FQs, moxifloxacin appears to be less affected by the bacterial efflux system because of its bulky C-7 substituents [49,50,51,52] and their optimized 8-methoxy substituent (Figure 4) [53,54].

**Figure 5:**
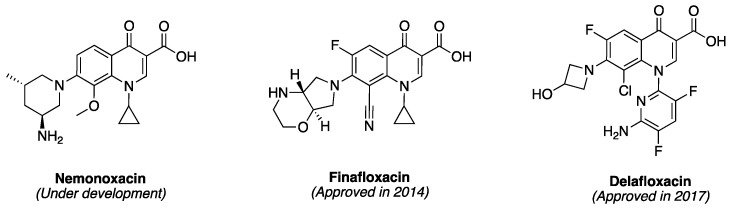
Structures of two recently approved quinolones and one in clinical development.

The newer generations FQs display potent activity against penicillin-resistant and multidrug-resistant (MDR) pneumococcus and anaerobes, while still retaining their activity against aerobes [55,56]. Several quinolones, most of which are FQs, are also currently at different stages of clinical development. For example, nemonoxacin (Figure 5) is a non-fluorinated broad spectrum quinolone (isothiazoloquinolone) that displays comparable in vitro Gram-negative activity as ciprofloxacin, levofloxacin, and moxifloxacin, but an enhanced potency against Gram-positive bacteria (including MRSA and MDR *S. pneumonia*e) [57]. It is currently under development for oral and intravenous treatment of community acquired pneumonia (approved in Taiwan), as well as the oral treatment of diabetic foot ulcer infections and skin and soft tissue infections [57]. Also, in 2014, finafloxacin (Figure 5) was approved by FDA as a topical otic suspension for the treatment of acute otitis eterna (swimmer’s ear), which is caused by susceptible strains of *P. aeruginosa* and *S. aureus* [58]. Delafloxacin (Figure 5) was approved in 2017 for the systemic treatment of acute bacterial skin and skin structure infections caused by a range of susceptible Gram-positive and Gram-negative bacteria (including ESKAPE pathogens) in adults [59]. The “ESKAPE” pathogens—encompassing *Enterococcus faecium*, *S. aureus*, *Klebsiella pneumonia*, *Acinetobacter baumanii*, *P. aeruginosa*, and *Enterobacter* spp—are responsible for many serious infections in hospitals [60]. Interestingly, unlike ciprofloxacin, moxifloxacin, and levofloxacin, which exhibit reduced activity at slightly acidic pH (5.0–6.5), finafloxacin and delafloxacin exhibit enhanced potency at this pH level, making them suitable for the eradication of *S. aureus* found in acidic environment.

**Figure 6:**
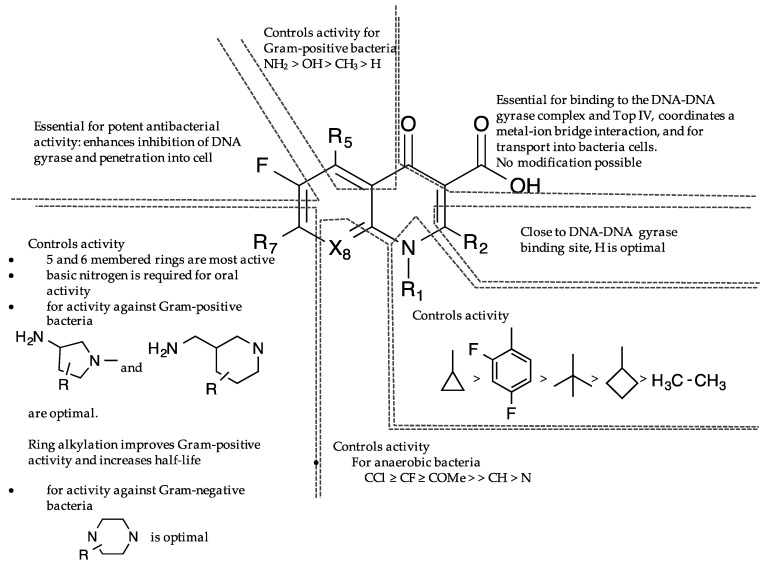
Structure-activity relationships of fluoroquinolones (adapted from reference [43]).

The comprehensive knowledge of the SAR of FQs (Figure 6) is central to the optimization of this class of drugs [43,61,62,63]. The pharmacophoric group of quinolones has been identified as a central bicyclic ring with hydrogen at position-2, a carboxyl group at position-3 and a keto group at position-4. This is known as the quinolone core (Figure 1) and it cannot be altered in any way without losing potency [43,64]. However, quinazolinediones (Figure 1) that share similar structural homology but lack the C3/C4 keto acid have now been demonstrated to be capable of overcoming quinolone resistance [65,66,67]. Mechanistic studies revealed that quinazolinediones overcome resistance via additional drug-enzyme contacts mediated by their “unusual” C7 substituent [5,65,66,67], and that substituents at position-7 greatly influence potency, spectrum, and safety of FQs [43]. These substituents are considered as the portion that bind to the subunit B of the DNA-enzyme complex, and changes at C-7 and C-8 of quinolones appear to play a significant role in the target preferences of this class of drugs [68].

## 4. Non-Classical Antiproliferative Activity of Fluoroquinolones

FQs were optimized and developed as antimicrobial agents, but several reports have shown that their potentials might be more than just antimicrobial actions [69]. Some FQs have been reported to display in vitro antiproliferative properties by inducing apoptosis, disrupting biochemical transformation of potentially cancerous cells, enhancing the uptake of other chemotherapeutic agents, and/or mediating immunomodulatory responses [70,71,72]. 

### 4.1. Ciprofloxacin

The antitumor efficacy of ciprofloxacin has been attributed to both intrinsic apoptosis and cell cycle arrest that could be reversed upon removal of the quinolone [12]. It was shown that ciprofloxacin induced a time- and dose-dependent growth inhibition, and apoptosis of various carcinoma, osteosarcoma, and leukemia cell lines [72]. The inhibition of mammalian cell growth by ciprofloxacin via the induction of tumor growth factor (TGF) β-1 by colonic epithelial cells was reported at concentration as low as 10 μg·mL^−1^ [12]. This is a clinically achievable concentration in human tissues using standard dosing regimen [12]. Moreover, of all of the different FQs that were tested in a cell free system, ciprofloxacin was the most potent inhibitor of mammalian DNA topoisomerase and polymerase [73]. Similarly, ciprofloxacin has been found to induce cell cycle arrest at the S and G2/M phases of androgen-independent carcinoma PC3, while sparing non-tumorigenic prostate epithelial cells [74]. This is particularly interesting because most chemotherapeutic agents that were used for treating advanced hormone resistant prostate cancer often result in 100% mortality, with a mean survival time of 7–8 months, as well as several associated toxicities [74].

### 4.2. Enoxacin

Enoxacin, a second-generation FQ, is one of the few FQs that retain the original 1,8-naphthyridone core of nalidixic acid. It has been used to treat bacterial infections ranging from gonorrhea to urinary tract infections [75] but has now been discontinued in the United States due to its severe side effects of insomnia and ability to trigger seizures or lower seizure threshold [76,77]. The human breast cancer cell line, MCF-7, was shown to be highly responsive to treatment with enoxacin, and growth inhibition was dose- and time-dependent, and irreversible in nature [78]. This is in contrast to ciprofloxacin, where its effect could be reversed upon the removal of the drug [12]. A separate study also showed that enoxacin was able to enhance the production of micro ribonucleic acids (miRNAs) that suppresses the tumor by binding to the miRNA biosynthesis protein, trans-activation response RNA-binding protein-2 (TRBP) [79]. MicroRNAs are small RNA molecules that regulate gene expression at the post-transcriptional level and are critical for many cellular pathways, with their disruption often being associated with the development of human tumors [80,81,82]. Unexpectedly, of the ten FQs that were analyzed, enoxacin was the only one that was capable of selectively stimulating miRNA expression with a median effective concentration of ~30 μM [83]. Importantly, enoxacin was found to be relatively non-toxic even at a high concentration of 150 μM [83], which is lower than its clinical dose [84]. The effect of enoxacin was also observed in vivo using a transgenic mouse line, and its miRNA-enhancing activity was TRBP-dependent [83]. Modifications and substitutions at the *N*-1, *C*-6, and *C*-7 positions of enoxacin significantly interfered with its miRNA-enhancing activity, suggesting that the compound forms a specific complex that is distinct from known targets of quinolones [83]. It also hints that the miRNA biogenesis-enhancing activity of enoxacin might probably not depend on the general FQ activities, but rather on the unique chemical structure of enoxacin. The cancer-selective properties of this molecule and its proposed cell cycle arrest of enhancing miRNA machinery could therefore represent a unique step towards the potential application of miRNA-based therapy in the treatment of human cancer [79].

### 4.3. Moxifloxacin

Moxifloxacin is a fourth-generation FQ antibiotic that differs mainly from ciprofloxacin, in that the piperazine moiety in ciprofloxacin has been replaced by a (1*S*,2*S*)-2,8-diazabicyclo[4.3.0]nonyl moiety. Moxifloxacin is also available in ophthalmic formulations that is widely used as prophylaxis for managing endophthalmitis after cataract surgery [85,86]. However, intraocular penetration of moxifloxacin was found to be inefficient for postoperative bacterial endopthalmitis, hence its use via an intracameral route of administration [87,88]. Based on this different route of drug delivery, the effect and safety of moxifloxacin on retinal ganglion cells (RGC5) of rats were examined. Moxifloxacin was found to exhibit both cytotoxic and anti-proliferative activity in vitro at a concentration >50 μg/mL [89]. Although a little higher than the expected prophylactic concentration of ≤50 μg/mL, an apparent lack of toxicity to other normal cellular activities [87,90] revealed the prospects of moxifloxacin to treat glaucoma patients with an increased risk of ganglion cell damage, where they may be used at such concentration advisedly.

### 4.4. Other Quinolones

**Figure 7:**
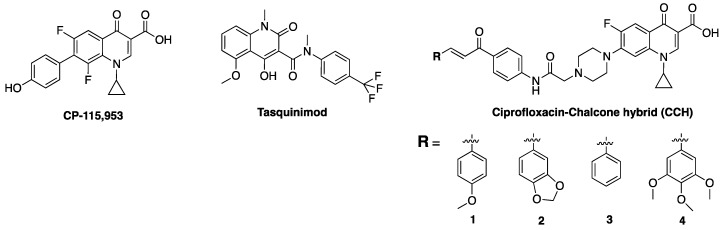
Structures of some fluoroquinolone-derivatives with enhanced antiproliferative activity.

Several novel tetracyclic FQs have been found to display anticancer properties against various human cells, such as breast cancer (MCF-7) and non-small cell lung cancer (A549), and were non-toxic to normal human-derm fibroblasts (HuDe) [68]. For example, 6,8-difluoroquinolones have been shown to be potent effectors of eukaryotic topoisomerase, as evident in the increased level of cleavage intermediates without impairing the DNA religation reaction of the enzyme [91]. One of the difluoro compounds examined, CP-115,953, (Figure 7) was twice as potent as etoposide at enhancing Top 2-mediated DNA cleavage in eukaryotic cells, while retaining potency against DNA gyrase [92]. Tasquinimod (Figure 7), a novel small-molecule inhibitor and second-generation oral quinolone-3-carboxamide, has also been reported to have anti-angiogenic properties and tumor growth-inhibiting activities against human prostate cancer. It is believed to mediate its activities via angiogenesis and immunomodulation, with a potency of about 30- to 60-times of its structural analog, linomide [93,94].

In summary, most FQs tend to induce nearly similar types of morphological alterations where cells become rounded, detached, and show cell membrane blebbing, a typical morphological change that signals the initiation of apoptotic processes [95].

## 5. Topoisomerases as Targets for Fluoroquinolone Actions: Prokaryotes versus Eukaryotes

**Figure 8:**
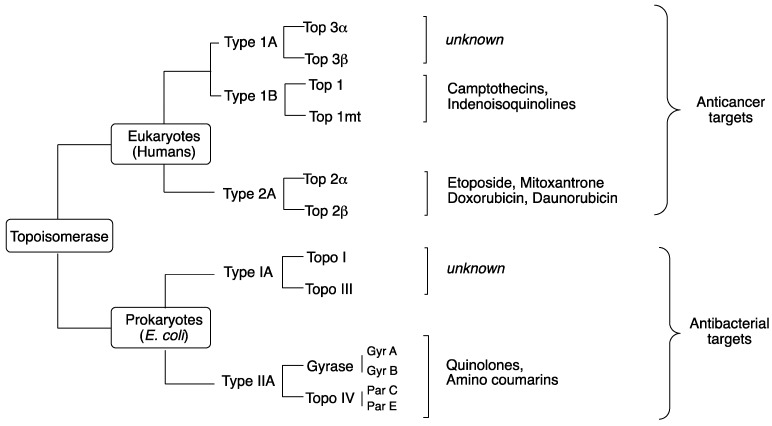
Classification of topoisomerases and their poisons.

Topoisomerases are large proteins found in humans and bacteria, with functional sizes of their enzyme-complex assembly ranging from 70 to 400 kDa [96]. Since the circumference of an average mammalian cell nucleus is almost one million times smaller than the length of the genome that needs to be packed into it, DNA compaction is necessary to squeeze these base pairs into the nucleus. Specifically, the entire genome of a single human cell (3 × 10^9^ base pairs, corresponding to approximately 1.8 m) needs to be squeezed into a nucleus with an average diameter of 6 μm [96]. Even the smaller circular *E. coli* genome (4.7 × 10^6^ base pairs) needs to be compacted within the nucleoid space of bacteria whose average circumference is 3000 times smaller [96]. The primary function of topoisomerases is therefore to introduce positive and/or negative supercoil during DNA transcription and replication to avoid super-helical tension and knots. Both human and bacteria topoisomerases are divided into type I and II, each with further subdivisions (Figure 8). Surprisingly, despite little or no sequence homology, both type IA and type IIA from prokaryotes and the type 2A enzymes from eukaryotes share structural folds that appear to reflect functional motifs within the critical regions of the enzymes [97]. Several excellent reviews have been published on DNA topoisomerases and their inhibitors [98,99,100,101,102]. FQs are safe in humans because they can discriminate between prokaryotic and eukaryotic topoisomerase targets. Whereas, gyrase and Top IV appear to be the preferential targets of FQs in Gram-negative and Gram-positive bacteria, respectively [69,103], other studies indicate that the primary targets of FQs are more drug-dependent than Gram-classification-dependent [104,105,106]. However, both prokaryotic enzymes can be targeted simultaneously due to the high degree of homology between them. Inhibition of eukaryotic Top 2 correlates with cytotoxicity in those cells, and clinically-relevant FQs are known to be safe and tolerated at concentrations that far exceed their cytotoxic threshold without bearing cytotoxic, genotoxic or carcinogenic potential. Since FQs also mediate antitumor activities in humans at clinically achievable concentrations, how they perform these extended roles is unclear.

### 5.1. Prokaryotic Topoisomerase Type IIA

In contrast to most anti-infective drugs, quinolones do not kill bacteria by interfering with a critical cellular process, rather, they corrupt the activities of two essential enzymes: DNA gyrase and Top IV, and subsequently induce them to kill cells by generating high levels of double-stranded DNA breaks [5,96,107,108,109,110,111]. Ordinarily, the relaxed bacterial DNA is too long to fit inside a bacterial cell, and must therefore be folded and compacted. Supercoiling (overwind or unwind) is an essential process during chromosome compaction, but also during transcription, replication, and DNA repair [111]. Due to the strain that is generated during unwinding, DNA gyrase transiently nicks each chromosomal domain, introduces a negative supercoil, and rapidly seals the nicked DNA before Top IV separates the linked daughter DNA molecules after replication. Both DNA gyrase and Top IV are classified as prokaryotic Top II (Figure 8), and they play two essential roles: (i) supercoiling (involved in chromosome replication) and (ii) decatenation (involved in chromosome partitioning such as topological resolution and topographical segregation) [96]. Crystal structures show that quinolones bind to the ternary complexes formed between Top II and the supercoiled DNA, hence, stabilizing a process that should have otherwise been very transient [108,109,110,112]. This stabilization effectively impedes the normal breaking-passing-resealing processes of the bacterial DNA by converting topoisomerases into physiological toxins. The extensive DNA fragmentation therefore becomes overwhelming for the bacterial repair mechanisms, thus leading to bacteria cell death [110,111].

Several theories have been propounded as the molecular basis by which quinolones interact with DNA-enzyme complex. One model proposes that quinolones interfere with DNA gyrase activity by selectively and directly interacting with the substrate DNA, rather than to gyrase [113,114,115]. This implies that the enzyme only plays a passive role in the inhibition mechanism [113]. However, the revised and widely accepted model is that quinolones do not bind directly to DNA, but to an enzyme-DNA complex [5,96,108,109,110,111]. The gyrase itself is believed to bind directly to the DNA, thereby creating a suitable pocket for quinolones to bind and stabilize the complex [116,117]. Stabilization of this ternary complex prolongs the normal transient nicking of the chromosome, thus leading to an extensive fragmentation of the DNA. The C3/C4 keto acid of quinolones (Figure 6) have long been known to chelate a divalent metal ion, but it was not until recently that this was captured in a crystal structure of a ternary *A. baumannii* Top IV-cleaved DNA-moxifloxacin complex [112]. The chelated Mg^2+^ ion appeared to be coordinated to four water molecules; two of which were situated close enough to Ser84 and Glu88 (equivalent of GyrA Ser83 and Glu87 in *E. coli*) to form hydrogen bonds [112]. The serine (Ser83 in *E. coli*) and acidic amino acid (Glu87 in *E. coli*) residues act as the anchor points that coordinate the bridge to the enzyme. Mutations at these highly conserved residues in the A subunit of Gyrase or Top IV are therefore the dominant mechanism of the resistance to FQs [5,64]. This supports the observation that water-metal ion interaction that bridges FQ to the enzyme plays a significant role in mediating quinolone activity [64]. The moxifloxacin binding model gives a structural explanation for key quinolone structure-activity relationships (Figure 6) [3] and the bulky substituent at position 7 of moxifloxacin was found to occupy a large and open pocket between the DNA and the ParE subunit [112]. This is consistent with the C7 position being the most versatile position for substitution in quinolones [44], suggesting that the basic substituent at this position possibly interacts with ParE/GyrB, thus explaining the effect of a ParE/GyrB mutation on quinolone sensitivity [118]. On the contrary, a recent crystal structure of gyrase-FQ cleaved complexes from *Mycobacterium tuberculosis* showed that moieties appended to the C7 position of FQs have no specific interactions with any Gyr B residue, but that the quinolone resistance-determining region (QRDR) of Gyr B provides a complementary van der Waals surface that can favorably accommodate fairly large moieties [119]. The exact orientation and interactions between FQs, DNA, and enzyme within the cleavage complex has however remained controversial [62,110,112,115,120,121].

### 5.2. Eukaryotic Topoisomerase Type IIA

Similar to prokaryotic topoisomerases, eukaryotic DNA topoisomerases control DNA topology and play an important role in the regulation of the physiological function of the genome and DNA processes such as replication, transcription, recombination repair, chromosome decondensation, and sister chromatid [122]. The human genome encodes six isoforms of topoisomerases, as opposed to four in *E. coli* (Figure 8). Type 1 enzymes cleave one DNA strand at a time, while type 2 cleave both strands to perform their catalytic functions [96]. Clinically used anticancer agents, such as camptothecins, target eukaryotic type 1B topoisomerases, whereas human type 2A topoisomerases are the targets of etoposide, anthracyclines (doxorubicin, daunorubicin), and mitoxantrone [96].

Since the major pharmacological breakthrough of identifying type 2A topoisomerase as the crucial target point of activity of etoposide and doxorubicin, targeting DNA Top 2 in cancer chemotherapy has been extensively researched towards the discovery of newer anticancer molecules [123,124,125]. In contrast to lower eukaryotes such as yeast and *Drosophila,* which encode a single type 2 enzyme [126,127], vertebrates have two isoforms of this enzyme, α and β. These two isoforms, which display similar enzymatic activities and share almost 70% amino acid sequence identity, are encoded by separate genes and differ in their physiological regulation and cellular functions. Top 2β is required for proper neurological development in mouse embryos and vertebrate cells can survive in their absence, while Top 2α is an essential enzyme primarily responsible for cell growth during DNA replication and mitosis [128,129,130].

Remarkably, clinically relevant quinolones, such as ciprofloxacin and moxifloxacin, display very little activity against human type 2 topoisomerases even at concentrations beyond their therapeutic doses [5]. The selectivity of FQs for bacterial topoisomerases was found to be 1000-fold more than human topoisomerases [131,132], although the basis for this has remained largely unexplained. However, unlike gyrase and Top IV, human Top 2α and 2β lack the serine and acidic amino acid residues present in prokaryotic Top II. Both of these residues are methionine in eukaryotic Top 2α isoform [5]. Since the primary interaction of FQs with bacterial type II enzymes is mediated through a water-metal bridge ion anchored by serine and acidic amino acid residues, the loss of the bridge anchors in Top 2α and 2β has been proposed as a possible underlying basis for discriminating between bacterial and human type II enzymes [5]. In effect, the human enzyme is the equivalent of a quinolone-resistant topoisomerase IV.

Anticancer drugs that target Top 2 (such as etoposide, doxorubicin, mitoxantrone, and ellipticine) generate DNA damage that interferes with crucial cellular processes by stabilizing the breaking-passing-resealing complex [10,11]. This lead to severe damage that overwhelms the repair pathways of the human cells, and that they are thus referred to as topoisomerase poisons [11,96]. The ability to specifically and selectively disrupt these pathways in cancer cells, but not in normal cells, leading to improved clinical responses, remains a challenge. DNA Top 2-targeting anticancer drugs are usually associated with secondary malignancies and non-specific tissue cytotoxicity [11]. How then do FQs mediate their activities in human cancer cells but not in normal cells?

### 5.3. Interfacial Inhibition of Topoisomerases by Fluoroquinolones

Whereas, Top 2β is expressed in both dividing and non-dividing cells, Top 2α is tightly linked to cell proliferation and is orders of magnitude higher in rapidly proliferating cells than quiescent cells [96]. Top 2α relaxes positively supercoiled DNA more efficiently than negatively supercoiled DNA, while Top 2β acts on both equally [133]. Drugs that target Top 2 are classified as either Top 2 poisons or Top 2 catalytic inhibitors. Top 2 poisons effectively block transcription and replication [11]. They interfere with Top 2 cleavage complexes (Top 2cc) by either inhibiting DNA religation (e.g., etoposide, doxorubicin) or enhancing the formation of Top 2cc (e.g., quinolone CP-115,953, ellipticines, azatoxins). However, drug-induced Top 2cc alone is not sufficient to rationalize the anticancer activity of Top 2 poisons as both normal and cancer cells express Top 2. For example, the cytotoxicity of etoposide was found to be decreased by RNA synthesis inhibitor [134], suggesting that it interferences between trapped Top 2cc and transcription might play a prominent role in cytotoxicity. On the other hand, Top 2 catalytic inhibitors do not interfere with crucial cellular processes and do not generate an increase in the levels of Top 2cc, rather, they are thought to kill cells through the elimination of essential enzymatic activities of Top 2 by inhibiting the ATP-driven energy transduction component, e.g., bisdioxopiperazines [11,135].

The mechanism of action of FQs in eukaryotic cancer cells is unknown, but studies have shown that the yeast Top 2 could be made sensitive to FQs, and that point mutations in eukaryotic Top 2 occurs in the region that is homologous to the QRDR of prokaryotic Gyr A [136]. Similarly, *E. coli* could be rendered sensitive to eukaryotic topoisomerase poisons by point mutations at Ser83 of Gyr A [137]. Typical eukaryotic Top 2 poisons (such as etoposide) exhibit significant toxicities against proliferating cells, but they also generate enzyme-mediated DNA damage [11]. Although the ability of FQs to target eukaryotic topoisomerases have been shown [13,14], it is unclear whether clinically used FQs (e.g., ciprofloxacin) indeed mediate their antiproliferative actions against cancer cells via topoisomerase interaction since they are relatively safe in human. Other reported mechanisms of action of FQs against rapidly proliferating cells (that spare non-tumorigenic cells), such as apoptosis and regulation of tumor growth factors [72,74], microRNA production [79,83], etc. suggest that intrinsic defects in DNA and checkpoints, which are landmarks of cancer cells [138], are likely the Achilles’ heels of cancer cells exposed to FQs. Physiologic and genetic changes in protein compositions/assembly in cancer cells, such as hyperactive DNA-enzyme machinery and their aggressively-proliferating nature, perhaps contribute to the more pronounced effect of clinically used FQs (such as ciprofloxacin) against cancer cells as opposed to normal cells. The mechanism of action of FQs on prokaryotic topoisomerases appears to be consistent with the mechanism of action of Top 2 poisons on eukaryotic topoisomerase. Indeed, some experimental FQ-derivatives (such as CP-115,953) have been shown to be as potent as etoposide at enhancing Top 2-mediated DNA cleavage in eukaryotic cells [92]. Note that these FQ analogs with significant activity against eukaryotic enzymes display similar toxicities that are associated with eukaryotic Top 2 poisons [13,14]. It is therefore unlikely that FQs with potent eukaryotic Top 2 activities will be useful as antibacterial agents going forward. The future of eukaryotic Top 2 as a drug target, as aptly discussed in an excellent review [11], will depend on whether isotype-specific agents can be developed. The ability of FQs to interact with eukaryotic Top 2β [13,14], impacting negatively on neurological development [129], is perhaps another reason why FQs should only be used in neonates and growing infants with great caution [139].

Based on this premise, many attempts have been made to modify antibacterial fluoroquinolones to produce novel antitumor agents [68,92,140,141,142,143]. A virtual screen of around 70,000 compounds revealed some quinolones to be potent inhibitors of human protein casein kinase (CK) 2 [144]. CK2 participates in the development of some type of cancers, and it is also implicated in viral infections and inflammatory failures [145], justifying some of the non-classical biological activities associated with FQs.

## 6. Immunomodulatory Properties of Fluoroquinolones

There is growing evidence that some antibiotics exert their beneficial effects not only by killing or inhibiting the growth of bacteria, but also indirectly by immunomodulation [146,147,148]. The first findings on the potential immunomodulatory properties of FQs were published in the late 1980s [149,150]. Whereas, the mechanism of antibacterial activity and effects of FQs on topoisomerase type II in prokaryotic and eukaryotic cells have been extensively investigated in vitro and in vivo, the mechanism underlying their immunomodulatory activities is still vague. However, possible cascade of intracellular processes leading to stimulatory or inhibitory effects on cytokines, chemokines, and other components of the immune system have been proposed [151,152].

The immune system can be thought of as a surveillance system that scans and ensures that tissues of the body are free of invading organisms and pathogens. When bacteria adhere to and colonize epithelial surfaces in their host macroorganism, they stimulate inflammatory responses and trigger the complex cytokine network. This is due to the released exoenzymes, exotoxins, polysaccharide, lipoteichoic and teichoic acid, peptidoglycan, and even DNA fragments, which are all proinflammatory [153,154]. One of the ways by which the immune system attack and get rid of foreign tissues/cells in such a complex environment is by induction of inflammation at the site of perturbation, which is by itself not a disease but a physiological response to a diseased state leading to the production of chemokines.

Chemokines (a large multifunctional family of cytokines; CHEMOtactic cytoKINES) are signaling proteins produced at the sites of infection or injury and act as chemoattractants to guide the migration of immune cells (such as lymphocytes) to their site of production [155]. Chemokines that are formed or produced under pathological conditions (such as infections and physical injury) are known as ‘inflammatory chemokines’, while those that are constitutively produced in certain tissues and that are responsible for leucocytes migration are known as “homeostatic chemokines”. The release of inflammatory chemokines is often stimulated by pro-inflammatory stimuli, such as interleukin-1 (IL-1), tumor necrosis factor (TNF)-α, lipopolysaccharides (LPS), or viruses, while homeostatic chemokines are produced and secreted without any need to stimulate their source cells [156,157].

FQs have been observed to interact with bacterial adherence to and colonization of epithelial surfaces, as well as alter the release of proinflammatory bacterial products [151,152]. Ciprofloxacin, moxifloxacin, levofloxacin, trovafloxacin, and grepafloxacin have all been shown to dose-dependently inhibit the synthesis of IL-1 and TNF-α at therapeutic concentrations in LPS-stimulated monocytes, while at the same time super-induce interleukin-2 (IL-2) in vitro [152]. Ciprofloxacin was seen to directly exhibit a concentration-dependent inhibition of LPS activity [158]. In vivo assessment of the effects of ciprofloxacin, rufloxacin, difloxacin, tremafloxacin, and trovafloxacin in a preclinical *Bacteroides fragilis* intra-abdominal infection model showed the elimination of this pathogen in 66% of treated animals at subtherapeutic doses [159,160,161,162,163,164]. FQs are inactive against *B. fragilis* in vitro, suggesting that their protective effect was not due to their antibacterial efficacy but probably due to the modulation of TNF production in vivo.

Moreover, the T-cell growth factor IL-2 belongs to the set of “early expressed” genes that are produced within few hours upon activation, and two to three days before initiation of cell division. Since this governs cell proliferation, it has been hypothesized that the IL-2 super-inducing activities of quinolones [150,165] enhance drug uptake during this crucial stage of cell division and ultimately modulate the rate of proliferation, as observed in the pulsing of phytohemagglutinin-stimulated peripheral blood lymphocytes (PBL) with ciprofloxacin [166].

The benefits and implications of these immunomodulatory properties are obvious and far-reaching. Inhibiting monokine synthesis, specifically IL-1 and TNF-α, could be advantageous in combating septicemia and septic shock where overstimulation of inflammation by LPS is a major virulence factor of hard-to-treat Gram-negative organisms. Moreover, the super-induction of IL-2 synthesis could be relevant in immunocompromised cancer patients that might need some form of external assistance for regulation of cell proliferation [151].

However, there appears to be a need for co-stimulants, trigger, or stress, to be applied to cells or experimental animals for FQs to mediate immunomodulatory actions. Administration of quinolones to intact animals or healthy volunteers, and/or in vitro exposure of various cells to quinolones alone did not exert, in general, any measurable immunomodulatory effect [151,152]. Thus, the potential therapeutic relevance of FQs should be interpreted with great caution, as it may be of relatively low importance compared to their intrinsic antibacterial activities.

## 7. Selectivity and Amplification of Desirable Properties in Fluoroquinolones

The SAR of FQs (Figure 6) is well known and all of the clinically relevant analogs have been optimized to suit antibacterial activities [3,43]. However, structural requirements and/or optimization for non-classical activities are also beginning to emerge. For instance, reports show that increasing the lipophilicity of FQs results in a commensurate improvement in antiproliferative efficacy [167]. This is consistent with published evidences that support the correlation between lipophilicity of compounds and antitumor efficacy [168,169,170,171,172,173,174]. Several derivatives of ciprofloxacin have been shown to display more potent in vitro antitumor activity than the parent compound, culminating into analogs whose inhibitory concentration (IC_50_) values are as low as ≤10 μM in various cancer cell lines [175]. Interestingly, novel *N*-4-piperazinyl-ciprofloxacin-chalcone hybrids (CCH) 1 and 2 (Figure 7) showed remarkable eukaryotic Top 2 activity that is comparable to etoposide, while CCH 3 and 4 displayed broad spectrum antitumor activity and high selectivity towards leukemia subpanel, respectively [167]. These show that lipophilicity plays a major role in the antiproliferative potentials of FQs.

Furthermore, fluoroquinolones with cyclopropyl moiety at position *N*1 (such as ciprofloxacin, sparfloxacin, and clinafloxacin) have been observed to exert increased production of interleukin 3 and granulocyte-macrophage colony-stimulating factor (GM-CSF) at clinically relevant dosing regimens, in contrast to those lacking the moiety [176]. GM-CSF stimulates stem cells to produce granulocytes (neutrophils, eosinophils, and basophils) and monocytes as part of the immune/inflammatory cascade crucial for fighting infections, and is clinically used to treat neutropenia in cancer patients undergoing chemotherapy, in AIDS patient during therapy, and in patients after bone marrow transplantation [177].

## 8. Limitations to the Development and Use of Fluoroquinolones

Early generation FQs, especially ciprofloxacin, have been very successful, but modifications that have led to optimization of pharmacokinetic properties and enhanced spectrum of activity have not been without their own costs. For example, specific idiosyncratic reactions have severely impeded the clinical relevance of agents such as trovafloxacin (hepatotoxic reactions) [178], temafloxacin (haemolytic uraemic syndrome) [179], grepafloxacin (cardiotoxicity) [180], clinafloxacin, and sitafloxacin (phototoxicity) [178,181], and in some cases led to their complete withdrawal from the market. It seems rather ironic that more quinolones have left the stage than remain. Nonetheless, the increasing knowledge of the interrelationship between SAR and ADR (adverse drug reactions) is poised to guide the future development of this class of drugs with well-informed predictions. Asides the unexpected ADRs, there are anticipated side effects of FQs that can be easily explained from their structure. The ketocarbonyl group at positions-3 and 4 with which they bind to the unpaired DNA bases via magnesium ions could potentially bind to other ions as well, such as aluminum, calcium, etc., as found in antacids, thereby forming a non-absorbable complex [182,183,184]. Also, the piperazine at position-7 has been associated with the tendency of FQs to displace γ-aminobutyric acid (GABA) or compete with its binding at the receptor sites within the central nervous system [185]. This suggests that FQs may only be used advisedly by patients with history of convulsion.

Furthermore, the clinical utility of FQs is threatened by multifactorial mechanisms of resistance present in almost every bacterial infection that is being treated with this class of drugs. Since the primary targets of FQs are DNA gyrase and Top IV, it is not surprising that the most prevalent resistance-conferring mutations occur at the highly-conserved serine and acidic residues in the A subunit of gyrase or Top IV (Ser83 and Glu87, respectively, in *E. coli*) [5,64]. This region is known as the quinolone resistance-determining region (QRDR) and alteration(s) in target protein structure alters the FQ-binding affinity of the enzyme. Other mechanisms of resistance to FQs include overexpression of multidrug resistance (MDR) efflux pumps, modifying enzymes, and/or target-protection proteins [186]. Surprisingly, long-term evolution experiments with *E. coli* showed that selection for fitness under some conditions can cause mutations within the genes that control supercoiling even in the absence of any antibiotic selective pressure [187]. This implicates evolution in the development of quinolone resistance, suggesting that merely restricting usage might probably not curtail long-term resistance development.

Lastly, while the immunomodulatory properties of FQs are beneficial in diverse conditions and are proven not to be an in vitro artifact, caution must be taken when used during transplantation. For instance, the super-induction of IL-2 (and potential activation of natural killer cells) and stimulatory effects on bone marrow generation by activation of IL-3 and GM-CSF synthesis could be important in immune-compromised cancer patients. On the contrary, this will be detrimental in patients transplanted with solid grafts on therapy with the immunosuppressive drug cyclosporine A, with whom additional T-cell stimulation would be deleterious upon rejection [151,188].

## 9. Conclusions

It is clear from the foregoing that the quinolone core is a privileged scaffold of drug that was accidentally discovered in the reaction flask of an observant chemist. From antimalarial to antibiotics, they evolved speedily into what has now become a reference towards the development of an “ideal” molecule [189,190]. Beyond the classical antimicrobial activities for which they were optimized and well-known for, FQs also display non-classical mechanisms of action, including but not limited to, antiproliferative and immunomodulatory properties. This provides a working scaffold that can either be developed as an anticancer or antibiotic agent. Unfortunately, the exact mechanism(s), by which they oscillate snugly between these extended roles is currently poorly understood. Since clinically relevant FQs are relatively safe in humans, their observed antiproliferative effects at clinically achievable concentrations might indeed be peculiar to aggressively-growing tumor cells, warranting a critical appraisal of their intrinsic potentials. Correlative and mechanistic studies of this class of drugs could perhaps give insights on their structural basis of selectivity for prokaryotic topoisomerases, and their ability to induce cell cycle arrest in eukaryotic cancer cells, while sparing non-tumorigenic ones. FQs that target eukaryotic topoisomerase show the same DNA damaging properties as other topoisomerase poisons [13,14], hence, drug optimization towards exclusive targeting of other eukaryotic mechanisms, such as apoptosis [74] and enhancement of miRNA production [83], will be a major step towards derivatizing this class of drugs exclusively for antiproliferative actions. How well FQs can be optimized for these evolving roles remain an interesting adventure in drug discovery. When fully understood, the intrinsic potentials of FQs could open a new paradigm in synthetic drug discovery, especially if the desired activity can be selectively amplified to discriminate bacteria from humans, and cancer cells from normal ones.

## Figures and Tables

**Table 1:** Comparison of fluoroquinolone generations (Adapted from refs [4,36,37]).

Generations	Microbiologic Activity	Administration and Characteristics	Indications
First generation Nalidixic acid, Cinoxacin (*Discontinued*), Flumequine	Enterobacteriaceae	Oral administration. Low serum and tissue drug concentrations. Narrow gram-negative coverage	Uncomplicated urinary tract infections Not for use in systemic infections
Second generation *Class I* Lomefloxacin (*Discontinued*), Norfloxacin, Enoxacin *Class II* Ofloxacin Ciprofloxacin	Enterobacteriaceae. Enterobacteriaceae, atypical pathogens; *Pseudomonas aeruginosa* (ciprofloxacin only), Pneumoccoci	Oral administration. Low serum and tissue drug concentrations. Improved gram-negative coverage, limited gram-positive coverage. Oral and intravenous administration. Higher serum, tissue, and intracellular drug concentrations, coverage of atypical pathogens	Uncomplicated urinary tract infections. Not for use in systemic infections. Complicated urinary tract and catheter-related infections. Gastroenteritis with severe diarrhea, prostatitis, nosocomial infections, sexually transmitted diseases
Third generation Levofloxacin, Sparfloxacin (Discontinued) Gatifloxacin (Discontinued)	Enterobacteriaceae, atypical pathogens, streptococci. Pneumoccoci MIC: 0.25–0.5 μg/mL	Oral and intravenous administration, similar to class II second-generation but with modest streptococcal coverage. Increased hepatic metabolism (sparfloxacin)	Similar indications as for second-generation. Community-acquired pneumonia in hospitalized patients or if atypical pathogens are strongly suspected
Fourth generation Trovafloxacin (Discontinued) Moxifloxacin Gemifloxacin	Enterobacteriaceae, *P. aeruginosa*, atypical pathogens, MSSA, streptococci, anaerobes, Pneumoccoci	Oral and intravenous administration. Similar to third-generation, but with improved gram-positive and anaerobic coverages	Consider for treatment of intra-abdominal infections
